# Intravenous Paricalcitol Versus Calcitriol for Secondary Hyperparathyroidism in Maintenance Hemodialysis: A Six-Month Single-Center Retrospective Cohort Study

**DOI:** 10.3390/jcm15145483

**Published:** 2026-07-13

**Authors:** Erman Özdemir, Erdoğan Özdemir, Pınar Özdemir

**Affiliations:** 1 Department of Nephrology, Pendik State Hospital, 34890 Istanbul, Turkey; 2 Department of Internal Medicine, Elazığ Fethi Sekin City Hospital, 23100 Elazığ, Turkey; erdoganozdemir@live.com; 3 Department of Nephrology, Kartal Dr. Lütfi Kırdar City Hospital, 34865 Istanbul, Turkey; pinarozdemir23@yahoo.com

**Keywords:** calcitriol, hemodialysis, paricalcitol, secondary hyperparathyroidism

## Abstract

**Background/Objectives:** Secondary hyperparathyroidism is a frequent component of chronic kidney disease-mineral and bone disorder in maintenance hemodialysis. Calcitriol and paricalcitol are used to suppress intact parathyroid hormone (iPTH), but the comparative advantage of paricalcitol in routine practice remains uncertain. This study compared 6-month biochemical PTH control in patients treated with intravenous calcitriol or intravenous paricalcitol. **Methods:** In this single-center retrospective cohort study, 312 adult hemodialysis patient files from 2023 to 2025 were screened. Patients who did not receive either intravenous study agent, switched between agents, or did not complete 6 months of treatment with the same agent were excluded. The final cohort included 185 patients, 100 treated with calcitriol and 85 with paricalcitol. The primary endpoint was target iPTH at 6 months, defined as 150–600 pg/mL. **Results:** Mean age was 58.9 ± 13.6 years, and 106 patients were male. Baseline iPTH was similar in the calcitriol and paricalcitol groups (684 ± 268 vs. 712 ± 291 pg/mL; *p* = 0.498). At 6 months, iPTH decreased significantly in both groups, with no significant between-group difference in follow-up iPTH (421 ± 214 vs. 398 ± 205 pg/mL; *p* = 0.456), percentage iPTH reduction (−36.8 ± 22.4% vs. −39.5 ± 24.1%; *p* = 0.431), or target iPTH achievement (62.0% vs. 60.0%; *p* = 0.781). Hypercalcemia and hyperphosphatemia rates were also similar. In multivariable analysis, treatment type was not independently associated with target iPTH achievement, whereas higher baseline iPTH and phosphorus were associated with lower odds of achieving target iPTH. **Conclusions:** Intravenous paricalcitol was not associated with superior 6-month PTH control compared with intravenous calcitriol in this real-world maintenance hemodialysis cohort. The findings indicate no demonstrated superiority of paricalcitol in this cohort and should not be interpreted as proof of pharmacological equivalence.

## 1. Introduction

Secondary hyperparathyroidism is a common component of chronic kidney disease-mineral and bone disorder (CKD-MBD) in maintenance hemodialysis. Progressive kidney failure leads to phosphate retention, reduced calcitriol synthesis, hypocalcemia, fibroblast growth factor 23 (FGF23) activation, and parathyroid gland hyperplasia. Persistent parathyroid hormone (PTH) elevation may contribute to high-turnover bone disease, vascular calcification, cardiovascular morbidity, and mortality. For this reason, treatment decisions are usually based on serial calcium, phosphorus, and PTH trends rather than a single laboratory value [1,2].

Vitamin D-based therapies are widely used to suppress PTH secretion in dialysis patients with persistently elevated or rising PTH. Calcitriol, the active form of vitamin D (1,25-dihydroxyvitamin D3), has long been part of secondary hyperparathyroidism treatment. Paricalcitol is a selective VDRA developed to suppress PTH while producing less intestinal calcium and phosphate absorption. Earlier comparative trials suggested fewer sustained calcium–phosphorus disturbances with paricalcitol, but later data have not consistently shown clear superiority over calcitriol [3,4].

Comparisons from clinical practice are useful because the choice between calcitriol and paricalcitol is affected by corrected calcium and phosphorus levels, dialysis vintage, phosphate binder and calcimimetic use, drug availability, reimbursement, cost, and physician preference. Randomized trials and meta-analyses comparing paricalcitol with calcitriol or other vitamin D-based therapies have produced mixed results [5,6,7,8]. We therefore compared 6-month PTH control in maintenance hemodialysis patients treated with intravenous calcitriol or intravenous paricalcitol for secondary hyperparathyroidism.

## 2. Materials and Methods

### 2.1. Study Design and Population

**Figure 1:**
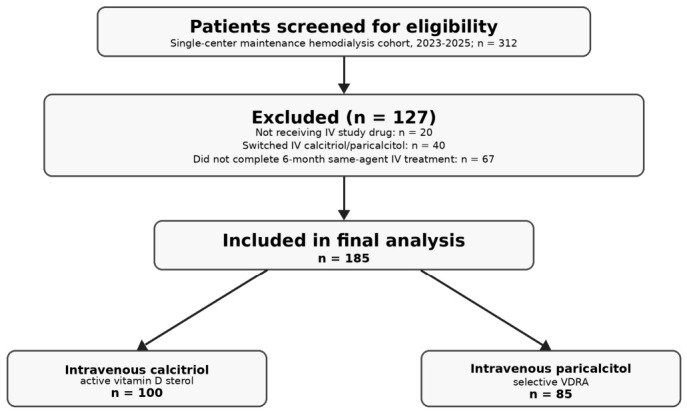
Patient selection flow diagram. Of 312 screened maintenance hemodialysis patient files, 127 were excluded because they were not receiving either intravenous study drug, switched between calcitriol and paricalcitol, or did not complete 6 months of treatment with the same intravenous agent. The final cohort included 185 patients: 100 treated with intravenous calcitriol and 85 treated with intravenous paricalcitol. IV, intravenous; VDRA, vitamin D receptor activator.

This single-center retrospective cohort study included adult patients with dialysis-dependent CKD stage G5D receiving maintenance hemodialysis and treatment for secondary hyperparathyroidism. Medical records from 2023 to 2025 were screened. Eligible patients received one of the two intravenous study agents (calcitriol or paricalcitol) and had adequate dialysis delivery, defined as single-pool Kt/V ≥ 1.4 and URR ≥ 70%. Of 312 assessed files, 127 were excluded and 185 patients were analyzed (100 calcitriol, 85 paricalcitol); the exclusion breakdown and patient flow are shown in Figure 1. No peritoneal dialysis patients were included.

Baseline was defined as the date of intravenous calcitriol or paricalcitol initiation or the first date on which all inclusion criteria were met. Follow-up was the biochemical measurement closest to month 6 within a ±4-week window. Data were collected from electronic medical records, hemodialysis charts, prescription and dialysis medication administration records, and routine laboratory reports. Patient selection is shown in Figure 1.

### 2.2. Inclusion and Exclusion Criteria

Patients were included if they were aged ≥ 18 years, had CKD G5D on maintenance hemodialysis with adequate dialysis delivery, had secondary hyperparathyroidism, received intravenous calcitriol or paricalcitol for at least 6 months, and had baseline and 6-month intact PTH, albumin-corrected calcium, phosphorus, Kt/V, and URR data.

Patients were excluded if they did not receive one of the two intravenous study agents, switched between agents, or could not complete continuous 6-month use of the same agent because of hypercalcemia, hyperphosphatemia, or non-adherence. Additional exclusions were primary or tertiary hyperparathyroidism, previous parathyroidectomy, active malignancy, kidney transplantation during follow-up, missing baseline or follow-up biochemical data, peritoneal dialysis, and new cinacalcet initiation during follow-up. Patients on stable cinacalcet at baseline (documented therapy without new initiation) were retained and adjusted for in the multivariable analysis.

### 2.3. Treatment Exposure and Biochemical Monitoring

Calcitriol and paricalcitol were prescribed intravenously and administered during or after hemodialysis sessions. Treatment choice was based on physician judgment, drug availability, reimbursement, and serial biochemical values, not random allocation. Dose titration followed PTH, albumin-corrected calcium, and phosphorus trends. Dose adjustments were not protocolized; they were made by the treating nephrologist during routine follow-up. Treatment was reduced or briefly interrupted for hypercalcemia, marked hyperphosphatemia, excessive PTH suppression, or a documented biochemical safety concern. Brief physician-directed interruptions followed by resumption of the same agent were recorded as dose-titration or safety events and were not counted as treatment switching. Because all included patients received intravenous therapy, administration route was not treated as a between-group confounder.

Phosphate binder exposure was recorded as any binder use and by predominant binder class. Binder categories were mutually exclusive and included calcium-based binders, sevelamer, lanthanum carbonate, and no binder use. Dialysate calcium concentration was recorded because it may affect corrected calcium–phosphorus balance and between-group comparability. Dialysate calcium concentration was missing in nine patients; these patients remained in the main analysis because primary endpoint data were complete. Serum calcium, phosphorus, alkaline phosphatase, albumin, and intact parathyroid hormone (iPTH) values were obtained from hospital records. Serum iPTH was measured by a chemiluminescent immunoassay using a UniCel DxI 800 analyzer (Beckman Coulter, Inc., Brea, CA, USA), with a laboratory reference interval of 22–74 ng/L. iPTH was reported in ng/L, which is numerically equivalent to pg/mL. Albumin-corrected calcium was calculated as follows: corrected calcium (mg/dL) = measured total calcium (mg/dL) + 0.8 × (4.0 − serum albumin (g/dL)). The 150–600 pg/mL range was used as a local clinical target consistent with the KDIGO approach of maintaining PTH at approximately 2–9 times the assay upper reference limit.

### 2.4. Outcomes

The primary endpoint was target PTH at 6 months, defined as follow-up intact PTH between 150 and 600 pg/mL. This interval was used as a pragmatic local target consistent with the KDIGO recommendation to maintain PTH at approximately 2–9 times the assay upper reference limit, not as a strict assay-specific calculation. Secondary endpoints were absolute PTH reduction, percentage PTH reduction, achievement of at least 30% and 50% PTH reduction, changes in albumin-corrected calcium and phosphorus, change in calcium–phosphorus product, hypercalcemia, hyperphosphatemia, treatment dose reduction, and temporary treatment interruption. Hypercalcemia was defined as albumin-corrected calcium > 10.2 mg/dL, and hyperphosphatemia as serum phosphorus > 5.5 mg/dL. All calcium analyses used albumin-corrected calcium.

### 2.5. Statistical Analysis

Continuous variables were reported as mean ± standard deviation or median with interquartile range according to distribution. Distributional assumptions were assessed using visual inspection of histograms and the Shapiro–Wilk test where appropriate. Categorical variables were summarized as number and percentage. Between-group comparisons were performed using the independent-samples *t*-test or Mann–Whitney U-test for continuous variables and the chi-square test or Fisher exact test for categorical variables, as appropriate. Fisher exact test was preferred when expected cell counts were small. Within-group changes were assessed with the paired-samples *t*-test for approximately normally distributed variables or the Wilcoxon signed-rank test for non-normally distributed variables.

Multivariable logistic regression was used to identify predictors of target PTH achievement. The model included age, sex, dialysis vintage, treatment type, baseline PTH, baseline corrected calcium, baseline phosphorus, phosphate binder use, and cinacalcet use based on clinical relevance and potential confounding. Results were reported as odds ratios (ORs) with 95% confidence intervals (CIs). Missing data were not imputed; the main analysis included patients with complete primary endpoint data. Additional sensitivity analyses were performed after excluding baseline cinacalcet users, restricting the analysis to patients with documented dialysate calcium concentration, evaluating patients with baseline PTH > 800 pg/mL, and evaluating patients with baseline hyperphosphatemia. A two-sided *p* value < 0.05 was considered statistically significant. Statistical analyses were performed using IBM SPSS Statistics for Windows, version 26.0 (IBM Corp., Armonk, NY, USA).

## 3. Results

### 3.1. Baseline Characteristics and Treatment Exposure

During the 2023–2025 study period, 312 maintenance hemodialysis patient files were screened at a single center. Only patients receiving one of the two intravenous study agents, calcitriol or paricalcitol, were eligible. Overall, 127 patients were excluded: 20 were not receiving either study drug intravenously, 40 switched between intravenous calcitriol and intravenous paricalcitol, and 67 had permanent discontinuation or prolonged interruption preventing continuous 6-month use of the same intravenous study agent because of hypercalcemia, hyperphosphatemia, or treatment non-adherence. The final cohort included 185 patients, 100 in the calcitriol group and 85 in the paricalcitol group (Figure 1). Because patients with permanent discontinuation or prolonged interruption were excluded, biochemical safety outcomes reflect events among patients who remained eligible for continuous 6-month treatment rather than complete treatment-emergent safety rates. Mean age was 59.7 ± 13.2 years in the calcitriol group and 57.8 ± 14.1 years in the paricalcitol group. Male sex was recorded in 58 patients (58.0%) receiving calcitriol and 48 patients (56.5%) receiving paricalcitol. Baseline demographic and biochemical variables were generally similar between groups. Mean single-pool Kt/V was 1.56 ± 0.08 vs. 1.55 ± 0.07 (*p* = 0.366), and URR was 79.1 ± 4.2% vs. 78.6 ± 4.0% (*p* = 0.409). Baseline PTH was 684 ± 268 pg/mL in the calcitriol group and 712 ± 291 pg/mL in the paricalcitol group, with no significant difference. Baseline characteristics and CKD-MBD treatment variables are summarized in Table 1; intravenous study-drug exposure and dose titration are presented in Table 2.

### 3.2. PTH Response

PTH decreased significantly in both treatment groups after 6 months (*p* < 0.001 within each group). In the calcitriol group, mean PTH declined from 684 ± 268 pg/mL to 421 ± 214 pg/mL. In the paricalcitol group, mean PTH declined from 712 ± 291 pg/mL to 398 ± 205 pg/mL. The absolute and percentage reductions in PTH were numerically greater with paricalcitol, but neither difference reached statistical significance. The proportions of patients achieving at least 30% or 50% PTH reduction were also similar between groups. Detailed PTH response outcomes are presented in Table 3.

### 3.3. Calcium–Phosphorus Metabolism and Biochemical Safety

Albumin-corrected calcium increased modestly in both groups, while mean follow-up corrected calcium values remained within clinically acceptable limits. At 6 months, corrected calcium was 9.18 ± 0.71 mg/dL in the calcitriol group and 9.05 ± 0.66 mg/dL in the paricalcitol group, without a statistically significant difference. Serum phosphorus showed a modest decrease in both groups. Hypercalcemia and hyperphosphatemia were numerically less frequent among paricalcitol-treated patients, but these differences were not statistically significant in categorical comparisons. Calcium–phosphorus metabolism and biochemical safety outcomes are summarized in Table 4. Because patients with permanent discontinuation or prolonged interruption were excluded from the analytic cohort, these categorical biochemical safety findings should be interpreted as events observed among continuously treated eligible patients rather than as complete treatment-emergent safety rates.

### 3.4. Sensitivity and Subgroup Analyses

Sensitivity analyses were performed to assess whether the main result was driven by baseline cinacalcet exposure, missing dialysate calcium documentation, severe baseline PTH elevation, or baseline hyperphosphatemia. Across these analyses, paricalcitol showed numerically greater PTH reduction in some strata, but no statistically significant advantage in target PTH achievement was observed. The sensitivity and subgroup analyses are summarized in Table 5.

### 3.5. Multivariable Analysis

A multivariable logistic regression model was constructed to evaluate independent predictors of target PTH achievement. After adjustment for age, sex, dialysis vintage, baseline PTH, baseline corrected calcium, baseline phosphorus, phosphate binder use, and cinacalcet use, treatment type was not independently associated with target PTH achievement. Lower baseline PTH and lower baseline phosphorus were independently associated with a higher likelihood of achieving target PTH at 6 months. The full multivariable logistic regression model is presented in Table 6.

## 4. Discussion

In this single-center retrospective cohort of 185 maintenance hemodialysis patients with secondary hyperparathyroidism, intravenous calcitriol and intravenous paricalcitol produced similar PTH control over 6 months. There were no statistically significant between-group differences in absolute PTH reduction, percentage PTH reduction, or target PTH achievement at 6 months (all *p* > 0.05; Table 3). Accordingly, calcitriol and paricalcitol provided comparable biochemical PTH control in this cohort. These results do not imply that the two agents are biologically identical; rather, this cohort did not show a significant 6-month biochemical advantage for paricalcitol over calcitriol.

These findings are in line with the randomized trial by Ong et al., in which oral paricalcitol and oral calcitriol had similar biochemical efficacy and safety in dialysis patients with secondary hyperparathyroidism. In that multicenter open-label trial, the proportion of patients achieving more than 30% iPTH reduction at 24 weeks was not significantly different between treatment arms, and hypercalcemia rates were comparable [5]. Our ≥30% PTH reduction rates (65.9% with paricalcitol and 67.0% with calcitriol) show the same direction, although our retrospective design and intravenous routine-practice dosing limit direct comparison.

Our findings also agree with the meta-analysis by Zhang et al., which compared paricalcitol and calcitriol in dialysis patients with secondary hyperparathyroidism and found no definite superiority of paricalcitol for the main efficacy and safety outcomes [6]. Xie et al. similarly concluded that the randomized evidence was insufficient to establish a clear comparative efficacy or safety advantage for paricalcitol over other vitamin D receptor activators in dialysis patients [7]. Broader comparative analyses have reported possible benefits of paricalcitol for iPTH reduction and survival-related outcomes, but they also noted uncertainty for corrected calcium–phosphorus endpoints and called for stronger prospective data [8].

A recent clinical-practice study by Murt reported a faster and greater PTH-lowering effect with paricalcitol than with calcitriol in hemodialysis patients, while also noting that both agents may maintain PTH within the desired range [9]. Earlier observational studies also suggested possible survival and hospitalization advantages among hemodialysis patients treated with paricalcitol compared with calcitriol, although these non-randomized outcome data remain vulnerable to residual confounding and should not be interpreted as proof of causal benefit [10,11]. In our cohort, PTH reduction did not differ significantly between the two agents, so our data do not confirm the between-group advantage suggested by some of these reports. Differences in baseline PTH severity, dose titration, adherence, dialysis vintage, phosphate binder exposure, cinacalcet use, and reimbursement-related prescribing may explain the discrepancy.

The corrected calcium–phosphorus results should also be considered. Early double-blind data from Sprague et al. suggested that intravenous paricalcitol could suppress PTH with fewer sustained episodes of hypercalcemia and elevated calcium–phosphorus product than calcitriol [3]. In our cohort, hypercalcemia, hyperphosphatemia, and the follow-up corrected calcium–phosphorus product did not differ significantly between groups (Table 4). These data therefore do not demonstrate a calcium–phosphorus safety advantage for paricalcitol over calcitriol.

The multivariable analysis suggests that baseline biochemical burden may matter more than the specific vitamin D-based treatment chosen. Treatment type was not associated with target PTH achievement after adjustment, whereas higher baseline PTH and higher baseline phosphorus reduced the likelihood of achieving target PTH. In practice, phosphate control, disease severity, and possible parathyroid gland autonomy may shape biochemical response. Switching from calcitriol to paricalcitol alone may therefore be inadequate in patients with severe hyperphosphatemia or very high baseline PTH unless phosphate binder therapy, dietary phosphate restriction, dialysis phosphate removal, calcimimetic use, and refractory hyperparathyroidism are also addressed.

These findings support individualized treatment. In patients with moderate PTH elevation and an acceptable corrected calcium–phosphorus profile, calcitriol remains a reasonable option. We did not perform a cost-effectiveness analysis, but the results may be relevant in settings where access and reimbursement affect the choice between calcitriol and paricalcitol. Paricalcitol may still be appropriate for selected patients with recurrent calcium–phosphorus abnormalities, calcitriol intolerance, or inadequate biochemical response, as suggested by studies of paricalcitol in calcitriol-resistant secondary hyperparathyroidism [12]. Related evidence from non-dialysis CKD stages 3–4 should be interpreted separately from maintenance hemodialysis cohorts but supports the broader need to evaluate treatment choice within the clinical context rather than by PTH reduction alone [13]. Our data, however, do not support routine replacement of calcitriol with paricalcitol in all maintenance hemodialysis patients solely to improve 6-month PTH control.

Several limitations should be noted. Patients with permanent discontinuation or prolonged interruption of intravenous calcitriol or paricalcitol therapy were excluded from the analytic cohort, so biochemical safety events may have been underestimated. Safety outcomes therefore reflect events among patients who remained eligible for continuous 6-month treatment, not a complete drug-safety analysis. The retrospective design carries a risk of selection bias and residual confounding. Treatment allocation was not randomized, and selection of calcitriol or paricalcitol may have been influenced by baseline biochemical profile, drug availability, reimbursement, physician preference, and previous treatment response. Although both agents were given intravenously, differences in dose titration, treatment intensity, adherence, and local prescribing patterns may have affected efficacy and safety. All patients met predefined dialysis adequacy criteria based on single-pool Kt/V ≥ 1.4 and URR ≥ 70%, but detailed dialysis-related variables such as delivered-dose variability, residual kidney function, dialyzer characteristics, and dietary phosphate intake were unavailable. Nutritional vitamin D status, phosphate binder dose and adherence, baseline cinacalcet dose stability, parathyroid gland size, bone-specific alkaline phosphatase, FGF23, vascular calcification, fracture, hospitalization, and mortality were not evaluated. Dialysate calcium concentration was unavailable in nine patients. Because no a priori sample size calculation was performed, the study may have been underpowered to detect modest differences, especially for biochemical safety outcomes. Follow-up was limited to 6 months; long-term clinical outcomes cannot be inferred.

Even with these limitations, the study adds practical data from daily hemodialysis care, where dose heterogeneity, confounding by indication, and biochemical endpoint interpretation are common. The single-center setting may have reduced variability in laboratory methods and routine management. Balanced baseline characteristics, inclusion of both efficacy and biochemical safety endpoints, sensitivity analyses, and multivariable adjustment support the internal consistency of the results. Prospective studies should standardize dose conversion, record adherence and phosphate binder intensity in detail, stratify patients by baseline PTH and phosphorus burden, and determine whether biochemical differences translate into patient-centered outcomes.

## 5. Conclusions

Among 185 maintenance hemodialysis patients with secondary hyperparathyroidism, intravenous calcitriol and intravenous paricalcitol achieved similar 6-month biochemical PTH control. No statistically significant between-group difference was observed for PTH reduction or for corrected calcium–phosphorus disturbances. Baseline PTH and phosphorus burden appeared to be stronger determinants of target PTH achievement than treatment type. Because this was not a non-inferiority trial, the findings indicate no demonstrated superiority of paricalcitol rather than proof of equivalence. Treatment should be individualized according to biochemical profile, patient characteristics, access, cost, tolerance, and the overall CKD-MBD strategy.

## Figures and Tables

**Table 1:** Baseline characteristics and CKD-MBD treatment variables.

Variable	Calcitriol Group, n = 100	Paricalcitol Group, n = 85	*p* Value
Age, years	59.7 ± 13.2	57.8 ± 14.1	0.348
Male sex, n (%)	58 (58.0)	48 (56.5)	0.834
Dialysis vintage, months	46.2 ± 31.5	49.8 ± 34.1	0.459
Single-pool Kt/V	1.56 ± 0.08	1.55 ± 0.07	0.366
URR, %	79.1 ± 4.2	78.6 ± 4.0	0.409
Diabetes mellitus, n (%)	42 (42.0)	29 (34.1)	0.272
Hypertension, n (%)	82 (82.0)	65 (76.5)	0.354
Cardiovascular disease, n (%)	31 (31.0)	27 (31.8)	0.911
Any phosphate binder use, n (%)	74 (74.0)	61 (71.8)	0.733
Calcium-based binder, n (%)	32 (32.0)	23 (27.1)	0.464
Sevelamer, n (%)	36 (36.0)	31 (36.5)	0.947
Lanthanum carbonate, n (%)	6 (6.0)	7 (8.2)	0.577
No phosphate binder, n (%)	26 (26.0)	24 (28.2)	0.733
Cinacalcet use, n (%)	18 (18.0)	16 (18.8)	0.885
Dialysate calcium concentration, n (%)			0.735
1.25 mmol/L	66 (66.0)	57 (67.1)	
1.50 mmol/L	28 (28.0)	25 (29.4)	
Not documented	6 (6.0)	3 (3.5)	
Baseline PTH, pg/mL	684 ± 268	712 ± 291	0.498
Baseline corrected calcium, mg/dL	8.74 ± 0.62	8.69 ± 0.58	0.578
Baseline phosphorus, mg/dL	5.42 ± 1.18	5.51 ± 1.23	0.614
Baseline Ca × P product	47.4 ± 11.6	47.9 ± 12.1	0.776
Baseline alkaline phosphatase, U/L	142 ± 76	151 ± 82	0.443
Albumin, g/dL	3.72 ± 0.41	3.69 ± 0.44	0.633

Note: All included patients were receiving maintenance hemodialysis and fulfilled predefined dialysis adequacy criteria based on single-pool Kt/V ≥ 1.4 and URR ≥ 70%. Single-pool Kt/V and URR are presented as mean ± standard deviation and were compared between groups. Phosphate binder categories refer to mutually exclusive predominant binder classes. Dialysate calcium was not documented in nine patients, but primary endpoint data were complete. For dialysate calcium, the *p* value refers to the overall distribution across 1.25 mmol/L, 1.50 mmol/L, and undocumented categories. CKD-MBD, chronic kidney disease-mineral and bone disorder; PTH, parathyroid hormone; Ca × P, calcium–phosphorus product.

**Table 2:** Intravenous study-drug treatment exposure and dose-titration profile.

Treatment Variable	Calcitriol Group, n = 100	Paricalcitol Group, n = 85	*p* Value
Starting weekly dose	0.75 ± 0.25 µg/week	6.0 ± 2.1 µg/week	Not compared
Mean maintenance weekly dose	1.20 ± 0.48 µg/week	9.2 ± 3.8 µg/week	Not compared
Patients requiring dose escalation, n (%)	42 (42.0)	35 (41.2)	0.910
Patients requiring dose reduction, n (%)	13 (13.0)	7 (8.2)	0.348
Temporary discontinuation, n (%)	5 (5.0)	3 (3.5)	0.728
Documented adherence ≥ 80%, n (%)	86 (86.0)	69 (81.2)	0.375

Note: IV, intravenous; VDRA, vitamin D receptor activator. Calcitriol and paricalcitol doses are presented descriptively and were not compared statistically because the agents are not directly dose-equivalent. Titration decisions were based on serial PTH, albumin-corrected calcium, and phosphorus values. Brief physician-directed temporary interruptions followed by resumption of the same agent were recorded as dose-titration or safety events and were not considered treatment switching. Adherence was based on dialysis medication administration records.

**Table 3:** PTH response at 6 months.

Outcome	Calcitriol Group, n = 100	Paricalcitol Group, n = 85	*p* Value
Baseline PTH, pg/mL	684 ± 268	712 ± 291	0.498
6-month PTH, pg/mL	421 ± 214	398 ± 205	0.456
Absolute PTH change, pg/mL	−263 ± 236	−314 ± 251	0.156
Percentage PTH change, %	−36.8 ± 22.4	−39.5 ± 24.1	0.431
≥30% PTH reduction, n (%)	67 (67.0)	56 (65.9)	0.872
≥50% PTH reduction, n (%)	35 (35.0)	31 (36.5)	0.835
Target PTH achievement, n (%)	62 (62.0)	51 (60.0)	0.781

Note: Negative values indicate reduction from baseline. Within-group PTH reduction was significant in both groups (*p* < 0.001). The *p* value column refers to between-group comparisons unless otherwise specified. PTH, parathyroid hormone. iPTH values are reported as pg/mL; ng/L and pg/mL are numerically equivalent for PTH.

**Table 4:** Calcium–phosphorus metabolism and biochemical safety.

Outcome	Calcitriol Group, n = 100	Paricalcitol Group, n = 85	*p* Value
Baseline corrected calcium, mg/dL	8.74 ± 0.62	8.69 ± 0.58	0.578
6-month corrected calcium, mg/dL	9.18 ± 0.71	9.05 ± 0.66	0.205
Corrected calcium change, mg/dL	+0.44 ± 0.59	+0.36 ± 0.55	0.350
Baseline phosphorus, mg/dL	5.42 ± 1.18	5.51 ± 1.23	0.614
6-month phosphorus, mg/dL	5.08 ± 1.09	4.96 ± 1.02	0.446
Phosphorus change, mg/dL	−0.34 ± 0.96	−0.55 ± 1.02	0.153
6-month Ca × P product	46.6 ± 10.8	44.9 ± 10.1	0.278
Hypercalcemia, n (%)	11 (11.0)	6 (7.1)	0.447
Hyperphosphatemia, n (%)	28 (28.0)	19 (22.4)	0.379
Treatment dose reduction, n (%)	13 (13.0)	7 (8.2)	0.348
Temporary treatment interruption, n (%)	5 (5.0)	3 (3.5)	0.728

Note: Hypercalcemia was defined as albumin-corrected calcium > 10.2 mg/dL, and hyperphosphatemia as serum phosphorus > 5.5 mg/dL. All calcium values represent albumin-corrected calcium. Ca × P, calcium–phosphorus product.

**Table 5:** Sensitivity and subgroup analyses for target PTH achievement.

Analysis	Calcitriol	Paricalcitol	*p* Value
Excluding baseline cinacalcet users: n	82	69	-
Excluding cinacalcet users: 6-month PTH, pg/mL	432 ± 218	407 ± 210	0.447
Excluding cinacalcet users: target PTH, n (%)	51 (62.2)	40 (58.0)	0.597
Documented dialysate calcium subgroup: n	94	82	-
Documented dialysate calcium subgroup: target PTH, n (%)	59 (62.8)	49 (59.8)	0.682
Baseline PTH > 800 pg/mL: n	38	33	-
Baseline PTH > 800 pg/mL: target PTH, n (%)	15 (39.5)	15 (45.5)	0.611
Baseline phosphorus > 5.5 mg/dL: n	46	39	-
Baseline phosphorus > 5.5 mg/dL: target PTH, n (%)	23 (50.0)	22 (56.4)	0.555

Note: All included patients were maintenance hemodialysis patients. The documented dialysate calcium subgroup excludes nine patients with unavailable dialysate calcium records.

**Table 6:** Multivariable logistic regression analysis for target PTH achievement.

Variable	Odds Ratio	95% CI	*p* Value
Paricalcitol vs. calcitriol	1.21	0.66–2.23	0.532
Age, per 1 year	1.01	0.98–1.03	0.487
Male sex	0.91	0.49–1.69	0.768
Dialysis vintage, per 12 months	0.96	0.86–1.07	0.441
Baseline PTH, per 100 pg/mL	0.82	0.73–0.93	0.002
Baseline corrected calcium, per 1 mg/dL	1.18	0.71–1.96	0.519
Baseline phosphorus, per 1 mg/dL	0.74	0.58–0.95	0.018
Phosphate binder use	1.09	0.55–2.17	0.803
Cinacalcet use	1.32	0.59–2.96	0.501

Note: The dependent variable was target PTH achievement at 6 months, defined as intact PTH 150–600 pg/mL. CI, confidence interval; PTH, parathyroid hormone.

## Data Availability

The data presented in this study are available from the corresponding author upon reasonable request, subject to institutional and ethical restrictions.

## References

[1] (2017). Kidney Disease: Improving Global Outcomes (KDIGO) CKD-MBD Update Work Group. KDIGO 2017 Clinical Practice Guideline Update for the Diagnosis, Evaluation, Prevention, and Treatment of Chronic Kidney Disease-Mineral and Bone Disorder (CKD-MBD). Kidney Int. Suppl..

[2] Jean G., Souberbielle J.C., Chazot C. (2017). Vitamin D in Chronic Kidney Disease and Dialysis Patients. Nutrients.

[3] Sprague S.M., Llach F., Amdahl M., Taccetta C., Batlle D. (2003). Paricalcitol versus Calcitriol in the Treatment of Secondary Hyperparathyroidism. Kidney Int..

[4] Hansen D., Brandi L., Rasmussen K. (2009). Treatment of Secondary Hyperparathyroidism in Haemodialysis Patients: A Randomised Clinical Trial Comparing Paricalcitol and Alfacalcidol. BMC Nephrol..

[5] Ong L.M., Narayanan P., Goh H.K., Manocha A.B., Ghazali A., Omar M., Mohamad S., Goh B.L., Shah S., Seman M.R. (2013). Randomized Controlled Trial to Compare the Efficacy and Safety of Oral Paricalcitol with Oral Calcitriol in Dialysis Patients with Secondary Hyperparathyroidism. Nephrology.

[6] Zhang T., Ju H., Chen H., Wen W. (2019). Comparison of Paricalcitol and Calcitriol in Dialysis Patients with Secondary Hyperparathyroidism: A Meta-Analysis of Randomized Controlled Studies. Ther. Apher. Dial..

[7] Xie Y., Su P., Sun Y., Zhang H., Zhao R., Li L., Meng L. (2017). Comparative Efficacy and Safety of Paricalcitol versus Vitamin D Receptor Activators for Dialysis Patients with Secondary Hyperparathyroidism: A Meta-Analysis of Randomized Controlled Trials. BMC Nephrol..

[8] Geng X., Shi E., Wang S., Song Y. (2020). A Comparative Analysis of the Efficacy and Safety of Paricalcitol versus Other Vitamin D Receptor Activators in Patients Undergoing Hemodialysis: A Systematic Review and Meta-Analysis. PLoS ONE.

[9] Murt A. (2025). Comparison of Parathormone Lowering Effects of Paricalcitol and Calcitriol in Hemodialysis Patients. World J. Nephrol..

[10] Teng M., Wolf M., Lowrie E., Ofsthun N., Lazarus J.M., Thadhani R. (2003). Survival of Patients Undergoing Hemodialysis with Paricalcitol or Calcitriol Therapy. N. Engl. J. Med..

[11] Dobrez D.G., Mathes A., Amdahl M., Marx S.E., Melnick J.Z., Sprague S.M. (2004). Paricalcitol-Treated Patients Experience Improved Hospitalization Outcomes Compared with Calcitriol-Treated Patients in Real-World Clinical Settings. Nephrol. Dial. Transplant..

[12] Tonbul H.Z., Solak Y., Atalay H., Turkmen K., Altintepe L. (2012). Efficacy and Tolerability of Intravenous Paricalcitol in Calcitriol-Resistant Hemodialysis Patients with Secondary Hyperparathyroidism: 12-Month Prospective Study. Ren. Fail..

[13] Coyne D.W., Goldberg S., Faber M., Ghossein C., Sprague S.M. (2014). A Randomized Multicenter Trial of Paricalcitol versus Calcitriol for Secondary Hyperparathyroidism in Stages 3-4 CKD. Clin. J. Am. Soc. Nephrol..

